# A Comparative Study on Changes in Total and Differential Milk Cell Counts, Activity, and Expression of Milk Phagocytes of Healthy and Mastitic Indigenous Sahiwal Cows

**DOI:** 10.3389/fvets.2021.670811

**Published:** 2021-06-21

**Authors:** Mohanned Naif Alhussien, Bibhudatta S. K. Panda, Ajay Kumar Dang

**Affiliations:** Lactation and Immuno-Physiology Laboratory ICAR-National Dairy Research Institute, Karnal, India

**Keywords:** mammary gland, SCC, DLC, macrophage, neutrophil, NETs

## Abstract

Milk somatic cell counts (SCCs) have been used as a gold standard to monitor mammary health as well as an indicator of raw milk quality. The present work was undertaken to compare the changes in the milk SCC, milk differential leukocyte counts (DLCs), phagocytic activity (PA) of milk neutrophils and macrophages (by nitroblue tetrazolium assay), extracellular trap formation (PicoGreen assay) and mRNA expression of various genes in milk neutrophils and macrophages (reverse transcription–polymerase chain reaction), and milk plasma cortisol concentration (enzyme-linked immunosorbent assay) in healthy, subclinical mastitis (SCM), and clinical mastitis (CM) cows. Milk was collected from healthy, SCM, and CM cows grouped based on their SCCs and California mastitis test with eight cows in each group. Milk SCC was estimated by SCC counter, and DLC was done after staining the milk slide under a microscope at 100×. Total SCCs in healthy, SCM, and CM cows were on an average of 128.30, 300.3, and 694.40 × 10^3^ cells/mL, respectively. Milk DLCs indicated a lower percentage of macrophage and lymphocytes and a higher (*p* < 0.05) percentage of neutrophils in SCM and CM compared to healthy milk. The percentage of mature segmented neutrophils was lower, whereas immature band neutrophils were higher (*p* < 0.05) in the SCM and CM groups as compared to healthy cows. The viability, *in vitro* PA, and extracellular trap formation of neutrophils were lower (*p* < 0.05) in SCM and CM milk samples as compared to healthy samples. However, the PA of macrophage remained unchanged in all the studied groups. The relative mRNA expression of Toll-like receptors (TLR2, TLR4), myeloperoxidase, and interleukin 2α (IL-2α) receptor (CD25) were minimum in healthy samples and increased (*p* < 0.05) with the progress of mammary inflammation. However, CD44 decreased (*p* < 0.05), and CD62L remained unchanged in mastitis as compared to healthy cows. Plasma cortisol concentrations were higher (*p* < 0.05) in mastitis as compared to healthy cows and were negatively correlated with the number of milk macrophages and the functions of milk phagocytes. Estimation of total SCC, milk DLC, and activity of milk phagocytes is essential for effective control and prevention of incidence of mastitis in dairy cows.

## Introduction

Mastitis, the inflammation of mammary gland, is one of the most costly and widespread diseases occurring in dairy cows worldwide (1, 2). The dairy sector, particularly in developing countries such as India, is facing massive economic losses because of mastitis, which can be attributed to reduced milk production; milk contamination with antibiotic residues; treatment, culling, or death of chronically infected cows; and many more (3, 4). Also, this disease possesses a serious zoonotic potential as it is associated with the shedding of various mastitis-causing microbes and their toxins in the infected milk (5). Although there is a number of pathogens that can cause mammary infection*, Staphylococcus aureus* is the most common bacteria that are responsible for chronic mastitis in dairy cows worldwide (6–8). This pathogen is exceedingly difficult to control by treatment alone. However, effective control can be gained through the prevention of new infections, which can be achieved by monitoring milk somatic and differential cell counts (9, 10).

Milk somatic cells (SCs) consist of milk-producing epithelial cells and leukocytes transmigrated to the mammary tissue. The count of somatic cell counts (SCCs) in milk is widely accepted as a novel method for the diagnosis of mammary infection in dairy cows (4, 11). Moreover, the proportions of various white cells in milk can give a deep insight into the severity and stage of mammary infection (12–14). Besides, the fluctuations in the proportions of various immune cells of milk during mammary infection may indicate an infection with specific groups of microbes and reflect the potential of mammary immune response (15–18). Milk phagocytes (neutrophils and macrophages) are the primary immune cells present in milk and represent the innate arm of mammary immune response during mammary infection (19, 20). Both neutrophils and macrophages perform phagocytosis, which is one of the fundamental mechanisms to ingest and intra-cellularly kill various invading pathogens during mammary infection (1, 21). Research in recent years revealed that neutrophils can also entrap and inactivate the invading pathogens by the release of extracellular traps (ETs), which composed mainly of DNA, histones, and many other antimicrobial proteins (22–24).

Sahiwal (SW) cows are considered one of the highest milk producers amongst the Indian native breed of cattle and are admirably adapted to the harsh climatic conditions of the tropics. Since these are high milk producers, they are regularly encountered with mammary infection (17, 21). Studying the changes in the milk somatic and differential leukocyte counts (DLCs) in healthy and mastitis cows and their impact on the cellular functions of the main arm of innate immunity (milk phagocytes) can provide critical information essential for effective control and treatment of mastitis. Therefore, the present study was designed to study and compare the milk SCC, DLC, and the cellular activity of milk phagocytes in term of phagocytosis and neutrophil extracellular trap (NET) formation in healthy, SCM, and CM SW cows naturally infected with *S. aureus*. An effort has also been made to investigate the changes in the expression of the key genes and receptors that mediate critical functions of neutrophils and macrophages.

## Materials and Methods

### Animal Selection, Sampling, and Management of the Cows

The approval of all the experiments carried out in this research work was obtained from the Animal Ethics Committee of the NDRI according to the Committee for the Purpose of Control and Supervision of Experiments on Animals rules, laid down by the Government of India. A total of 24 indigenous SW cows were selected from the Livestock Research Centre (LRC) of the National Dairy Research Institute, Karnal, India. All the experimental cows were high yielder (>10 kg/d) and in their early stage of lactation (days in milk <90). The cows were multiparous with an average body condition score of 3.5. Evaluation of the health status of mammary gland and classifying cows into three groups of healthy (*n* = 8), subclinical mastitis (SCM) (*n* = 8), and clinical mastitis (CM; *n* = 8) was done based on several diagnostic tests such as California Mastitis Test (CMT), milk SCC, and electrical conductivity (EC). The groups were classified as follows: healthy group (CMT score = 1, EC = 5.90 mS/cm, and SCC <200 × 10^3^ cells/mL), SCM group (CMT score = 2, EC = 6.25 mS/cm, and SCC = 200–500 × 10^3^ cells/mL), and CM group (CMT score = 3, EC = 7.20 mS/cm, and SCC >500 × 10^3^ cells/mL). Also, several other clinical symptoms of clinical mammary infection were considered during the selection of CM group such as swelling and pain in the infected quarters, fever, and abnormal alteration in milk characteristics including changes in the color, consistency, and blood in milk. Three milk samples were collected from each animal at 1-day intervals. Composite milk samples representing all four quarters were collected from healthy cows. However, the milk samples were collected from the affected quarters only in the case of SCM and CM groups. The aforementioned tests were repeated daily to make sure the health status of mammary gland is still the same. These cows were fed with *ad libitum* green fodder and concentrate diet (20% crude protein and 70% total digestible nutrient) as per practices followed in the LRC-NDRI for early lactating cows. Fresh and clean tap water was provided at all times of the day.

### Estimation of Somatic and DLCs of Milk

Several diagnostic tests such as CMT, milk SCC, and EC were performed to classify the experimental cows into various groups based on their udder health as described by Alhussien and Dang (25). For the CMT procedure, 2 mL of milk sample was loaded in the CMT paddle kit and mixed with 2 mL of CMT reagent (DeLaval Pvt. Ltd., India) in each cup. The mixture of both milk sample and the reagent was rotated for <1 min, and the result was recorded as follows: score 1 (negative, the mixture remains unchanged), score 2 (trace or slight to distinct gel formation), and score 3 (strong and clear gel formation). The measurement of SCC and EC in milk samples was performed using a Lactoscan milk analyzer (Milkotronic Ltd., Stara Zagora, Bulgaria), which utilize a digital system to measure the milk concentration of the fluorescent cells as described by Alhussien and Dang (4). Counting of milk SCC was also crosschecked microscopically as described by Panda et al. (16). Briefly, 96% ethyl alcohol was used for fixation (3 min), xylene was used to remove fat (10 min), and methylene blue dye for staining (15 min). Differential leukocyte counting (DLC) of milk was done to estimate the percentage of various immune cells secreted in milk, including macrophages, neutrophils, and lymphocytes as per the method described by Dang et al. (26). Using the same SCC smear, milk DLC of various immune cells (macrophages, neutrophils, and lymphocytes), and the type of neutrophil (mature with segmented nuclei and immature with band nuclei), staining was carried out in 30 fields under oil immersion at 100× (Olympus IX51 microscope): DLC of a particular leukocyte (%) = (no of that specific leukocyte / total no of leukocytes) × 100.

### Mastitis-Causing Bacteria

Milk samples obtained from healthy, SCM, and CM groups were studied for the presence of possible mastitis-causing bacteria including the major organisms mainly responsible for mammary infection in Indian breed of cattle under tropical conditions. These include *S. aureus, Streptococcus agalactiae*, and *Escherichia coli*, which were studied by pour plate method using Baird–Parker agar, blood agar, and eosin–methylene blue agar as described by Alhussien and Dang (17). Briefly, the milk samples were incubated at 37°C for 24–48 h until the appearance of colonies. Characterization of the isolates was carried out using negative staining and Gram staining. Gram-positive bacteria (*S. aureus* and *S. agalactiae*) were distinguished and confirmed by several approaches including cellular arrangement, catalase production, Hotis test, and coagulase test. More than 50% of the milk samples isolated from SCM and CM cows were positive for *S. aureus*. Milk samples that were positive for other pathogens such as *S. agalactiae* and *E. coli* were excluded. Milk samples that were positive only for *S. aureus* were selected for further processing. Milk samples collected from healthy cows did not display any bacterial content and were completely free of infection.

### Isolation of Milk Phagocytes

The isolation of milk phagocytes (neutrophils, macrophages) was carried out using the gradient density method as described in our earlier studies (17, 27). Briefly, milk sample (200 mL) was centrifuged, and the obtained cell pellet was washed and suspended in Dulbecco phosphate-buffered saline (PBS, Himedia, India Pvt. Ltd.) containing 0.5 mg/mL gelatin. For milk neutrophil isolation, 3 mL each of Histopaque 1077 and Histopaque 1119 (Sigma–Aldrich, St. Louis, MO, USA) was carefully layered above each other, followed by layering the leukocyte suspension (3 mL) over the Histopaque 1077. Centrifugation (2,000 × *g*, 20 min, 4°C) was carried out, and the milk neutrophils were harvested at the interface of the Histopaque 1119 and Histopaque 1077 layers. For milk macrophage isolation, the leukocyte pellet was dissolved in 5 mL of Dulbecco PBS, and the cell suspension was layered on 4 mL Histopaque 1083 (Sigma–Aldrich). Centrifugation was then carried out (300 × *g*, 15 min, 4°C), and milk macrophages were harvested at the interface of PBS and the Histopaque 1083. The obtained milk phagocytes (neutrophil/macrophage) were washed with PBS and suspended in RPMI media (Sigma–Aldrich) for further processing. The number and viability of milk phagocytes were evaluated by trypan blue method using hemocytometer (Reinfeld, Germany). The purity of milk phagocytes was >96% as evaluated by May–Grünwald–Giemsa staining under oil immersion at 100× (Olympus IX51 microscope).

### Phagocytic Activity of Milk Neutrophils and Macrophages

The phagocytic activity (PA) of milk phagocytes was estimated under *in vitro* conditions using nitroblue tetrazolium (NBT) assay as described in our earlier studies (17, 27). Briefly, RPMI-1640 media was used to adjust the number of milk neutrophils/macrophages to 5 × 10^6^/mL in a flat-bottomed tissue culture plate (Coster; Sigma–Aldrich, USA). The milk phagocytes cells were stimulated using zymosan and NBT (Sigma–Aldrich, USA), followed by an incubation step for 3 h in a humidified CO_2_ incubator (37°C, 95% air and 5% CO_2_). Finally, the optical density (OD) was measured at 540 nm by an enzyme-linked immunosorbent assay (ELISA) reader (MutiSkan GO; Thermo Scientific, Finland).

### Quantification of Milk NETs

The release of extracellular traps from milk neutrophils was estimated by PicoGreen, a DNA-binding dye (Invitrogen; Thermo Scientific, USA) as described earlier (24, 28). For NET study, RPMI-1640 medium lacking phenol red and having 2% fetal bovine serum (Thermo Scientific, USA) was used to suspend the isolated milk neutrophils. Approximately 2 × 10^5^ live cells of milk neutrophils/well were seeded (in triplicate) in a 96-well flat-bottomed tissue culture plate (Coster; Sigma–Aldrich, USA) using the aforementioned RPMI-1640 medium. The milk neutrophils of healthy, SCM, and CM animals were incubated with zymosan (1 mg/mL) in 200-μL volume (3 h, 37°C, 5% CO_2_). Micrococcal nuclease (5 U/well; Sigma–Aldrich, USA) was then added and incubation (10 min) was done followed by centrifugation (700 × *g*, 5 min). Thereafter, 100 μL/well of supernatant was transferred into 96-well flat-bottomed black polystyrol microplates (Greiner Bio-one, Germany). PicoGreen reagent (Invitrogen; Thermo Scientific, USA) was diluted (1:200) in 10 mM Tris base buffered with 1 mM EDTA and then added 100 μL/well followed by an incubation step for 5 min in the dark. Automated multiplate reader was used to estimate the formation of NETs in arbitrary fluorescence units (AFU) at excitation and emission wavelengths of 480 and 525 nm, respectively. Unstimulated milk neutrophils suspended in RPMI medium lacking phenol red were used as negative controls. To prove the DNA nature of extracellular traps of neutrophils, 90 U of DNase I (Sigma–Aldrich, USA) was added to the culture of milk neutrophils and zymosan approximately 15 min prior to the completion of the incubation period.

### Real-Time Gene Expression Study

Total RNA was isolated from milk neutrophils and milk macrophages using RNeasy Mini Kit (Qiagen, India Pvt. Ltd.). DNase 1, RNase-free (Qiagen, India Pvt. Ltd.) was used to eliminate the genomic as per the manufacturer's protocol. Agarose gel (1.6%) electrophoresis was used to assess the integrity of RNA samples, and the purity of RNA samples was assessed using Biospec-nano Spectrophotometer (Shimadzu Corp., Kyoto, Japan) based on the OD absorption ratio at $\overset{\lambda \text{260/}\lambda }{280}$ in which the ratio of 2 was considered as pure RNA. The isolated RNA samples were then stored at −80°C until further processing. An amount of 1 μg for each RNA sample was reverse transcribed into cDNA in a 20 μL reaction mixture using cDNA synthesis kit (Thermo Scientific, USA) as per the manufacturer's protocol. Primers of housekeeping genes (β-actin, GAPDH), Toll-like receptors (TLR2, TLR4), myeloperoxidase (MPO), and the cluster of designation receptors (CD25, CD44, CD62L) were adapted from the available literature and are shown in Table 1. The relative mRNA expression of TLR2, TLR4, and MPO was studied in both neutrophils and macrophages. However, the relative expression of CD25, CD44, and CD62L was studied only in neutrophils. SYBR Green Master Mix kit (Thermo Scientific, USA) was used to amplify the cDNA samples in a Roche Light Cycler 480 instrument following the protocol described earlier (17, 27). A no-template control was included, and the data were normalized using two housekeeping genes, GAPDH and β-actin. The healthy control group of cows was taken as a calibrator for which the relative mRNA expression of all the studied genes was estimated. The 2^−ΔΔCT^ method was used to calculate the relative quantification of the studied genes in milk phagocytes as described elsewhere (29).

**Table 1:** Details of various primers used in the study.

**Genes**	**Sequence (5^**′**^ → 3^**′**^)**	**Accession no**.	**Size (bp)**	**Annealing temperature (^**°**^C)**
TLR2	F: GCCTTGACCTGTCCAACAAT R: GACCTGAACCAGGAGGATGA	NM174197.2	199	59
TLR4	F: GGCATCATCTTCATCGTCCT R: CTGGACTCTGGGGTTTACCA	AY634630.1	178	59
MPO	F: TCGATACCAACCTATGCAGCCCAA R: ATTTGGTTCTGGCGGTTCAGCTTC	NM_001113298.1	147	59
CD25	F: ACATCGGCAGTGGTCTCAG R: GAACCTCCACATCAGCAAGC	NM_174358.2	60	58
CD44	F: CTGTCAACAGTAGGAGAAGGTGTG R: TCCTCCATGGTTCCATTCCCATTG	NM_174013.3	73	58
CD62L	F: TCCAGAACCAACCTGTCGAGTG R: TCCATGGTTCCCAAATCGGGTTC	NM_174182.1	66	58
GAPDH	F: GGGTCATCATCTCTGCACCT R: GGTCATAAGTCCCTCCACGA	NM_001034034	176	59
β-Actin	F: CATCGCGGACAGGATGCAGAAAGC R: GCGCGATGATCTTGATCTTCATTG	NM_173979.3	71	58

*F, forward; R, reverse; TLR2 and TLR4, Toll-like receptors; MPO, myeloperoxidase; CD25, CD44, CD62L, cluster of designation molecules; GAPDH and β-actin, housekeeping genes*.

### Quantification of Plasma Milk Cortisol

Plasma milk cortisol was quantified in the skimmed milk as per the method described elsewhere (27, 30). The concentrations of plasma cortisol were estimated using a competitive format of bovine cortisol hormone ELISA kit (Cusabio Biotech Co., Ltd.) as per the manufacturer's protocols. The minimum detectable dose of cortisol was <0.049 ng/mL, and the detection range was 0.049–200 ng/mL. The intra-assay and interassay coefficients of variance were <8 and <10%, respectively.

### Statistical Analysis

The analysis of the data was performed by repeated-measures one-way analysis of variance using SAS (version 9.1; SAS Institute Inc., Cary, NC, USA). The pairwise comparison was performed using the Tukey multiple-comparisons test. To analyze the correlations between milk SCC and various studied parameters, SAS 9.1 was used to calculate Pearson correlation co-efficients and the corresponding *P* values; *p* < 0.05 was considered statistically significant.

## Results

### Milk SCC and DLCs

**Figure 1:**
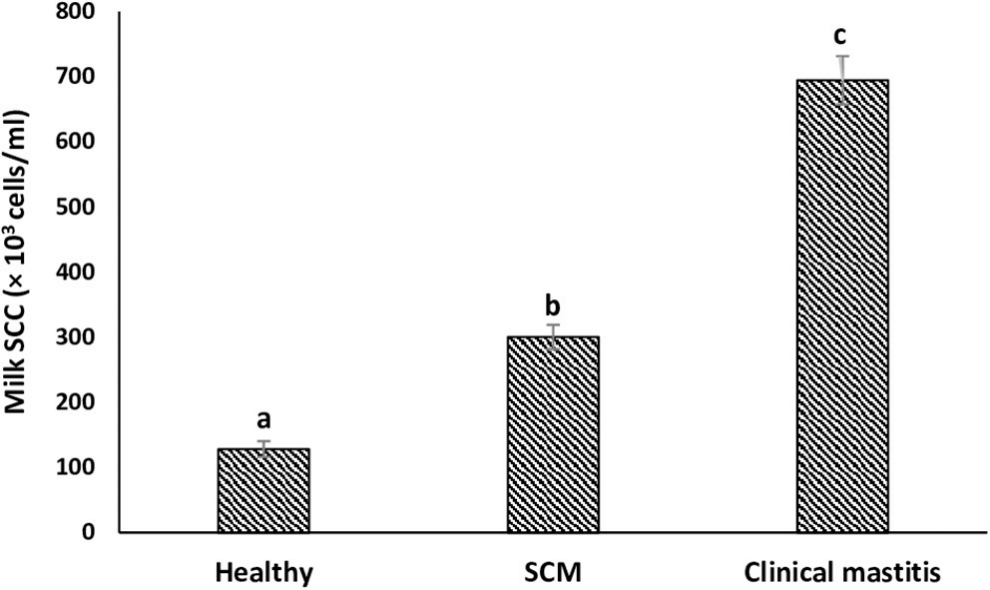
Milk somatic cell count (SCC) in healthy, subclinical mastitis (SCM) and clinical mastitis Sahiwal cows. Data were expressed as mean ± SEM. Means statistically (*p* < 0.05) different between the groups are shown with superscript letters (a–c).

**Figure 2:**
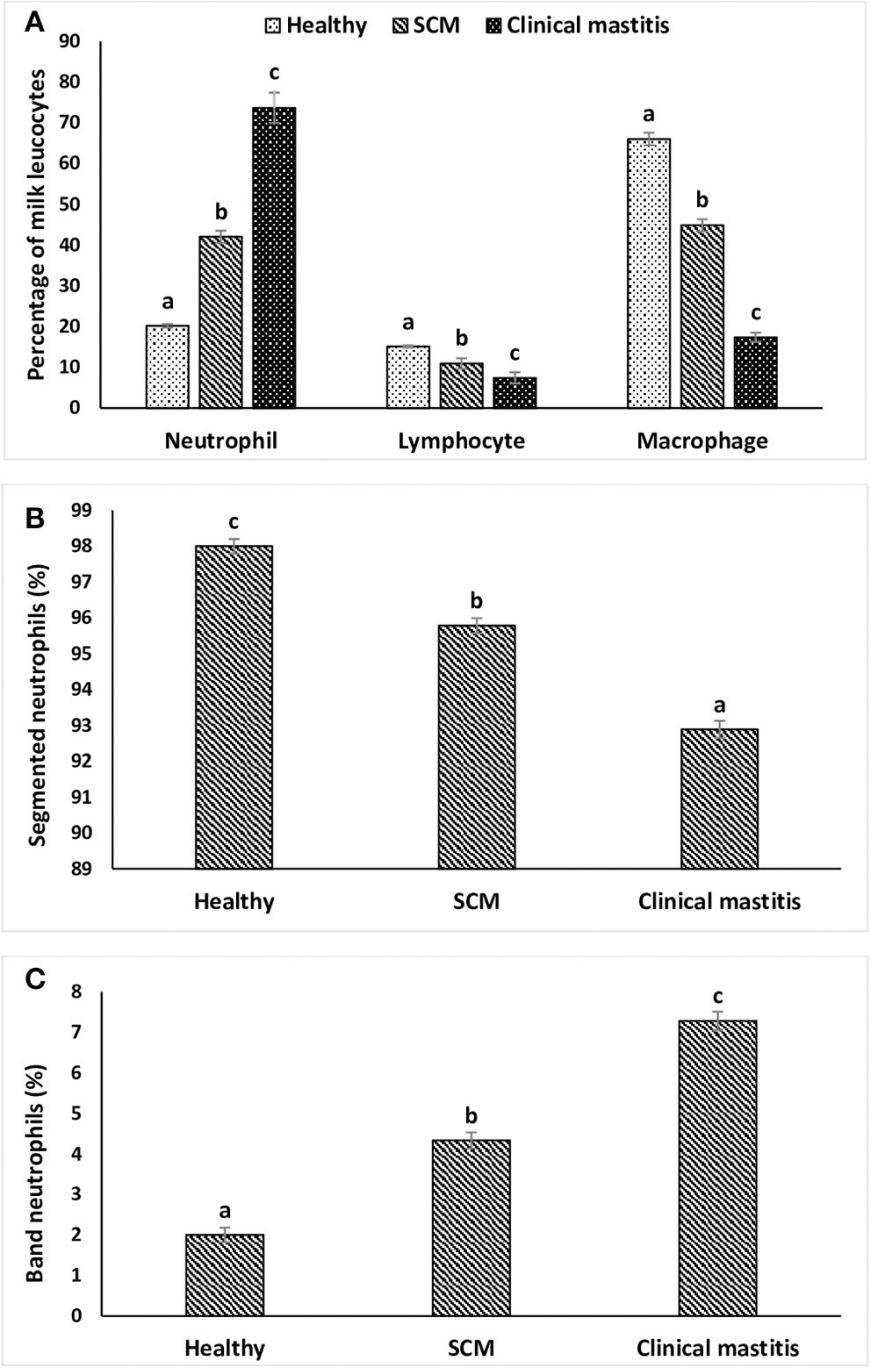
Differential milk leukocyte counts. **(A)** The percentage of milk neutrophils, macrophages, and lymphocytes; **(B)** segmented neutrophils; **(C)** band neutrophils in healthy, subclinical mastitis (SCM), and clinical mastitis Sahiwal cows. Data were expressed as mean ± SEM. Means statistically (*p* < 0.05) different between the groups are shown with superscript letters (a–c).

**Figure 3:**
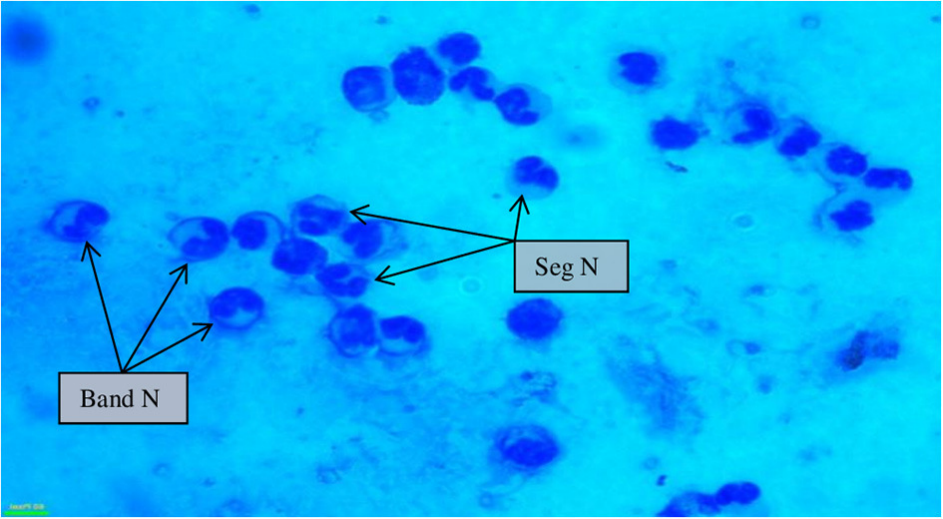
Milk somatic cell counts in mastitis cows (100×) showing a maximum influx of banded neutrophils along with segmented neutrophils during mastitis. Seg N, segmented neutrophils; band N, banded neutrophils.

SCs were counted in healthy animals and animals diagnosed with SCM and CM. The number of SCs (× 10^3^ cells/mL) in healthy samples of milk was 128.30 ± 12.10, which were found to be significantly increased (*p* < 0.05) in samples of milk from animals with SCM, and the highest values were recorded in milk collected from the CM group (694.40 ± 37.20) (Figure 1). The percentage of milk neutrophils was 20.16 ± 0.40% in healthy milk increased (*p* < 0.05) in SCM (42.01 ± 1.50) and attained the maximum values in the CM group (73.66 ± 3.73) (Figure 2A). Milk macrophages and lymphocytes displayed different patterns compared to milk neutrophils in which they were highest in the healthy group and decreased (*p* < 0.05) with the progress of mammary infection. Macrophage was the dominant leukocyte in healthy milk (macrophage = 66.03% vs. neutrophil = 20.16%), both macrophage and neutrophils were dominant in SCM milk (macrophage = 44.84% vs. neutrophils = 42.01%). However, neutrophils represent the major leukocytes in milk collected from the CM group (neutrophils = 73.66% vs. macrophage = 17.39%) (Figure 2A). The percentage of the mature form of milk neutrophils (segmented type) was maximum in healthy milk (98 ± 0.20) and decreased significantly (*p* < 0.05) with the progress of mammary inflammation, with the lowest values recorded in the CM group (92.89 ± 0.24) (Figure 2B). However, the immature type (band neutrophils) followed an inverse pattern of that of segmented neutrophils in which it was lowest in healthy milk (2.0 ± 0.18) and increased significantly (*p* < 0.05) in mastitis milk (7.28 ± 0.23) (Figures 2C, 3). Milk SCC was positively correlated (*p* < 0.05) with the percentage of milk neutrophils and the immature band type of neutrophil, as well as the concentration of milk plasma cortisol. However, it was negatively correlated (*p* < 0.05) with the percentage of milk macrophage, segmented neutrophils, and the PA of neutrophils (Table 2).

**Table 2:** Correlation of milk SCC with milk DLC, phagocytic activity (PA) of phagocytes, NET formation, and plasma cortisol in healthy and mastitis cows.

**Parameters**	**SCC**	**PMN**	**Seg PMN**	**Band PMN**	**L**	**M**	**PA of PMN**	**PA of M**	**NETs**	**Cortisol**
SCC	1									
PMN	0.89^*^	1								
Seg PMN	−0.86^*^	−0.91^*^	1							
Band PMN	0.84^*^	0.92^*^	−0.89^*^	1						
L	−0.82^*^	−0.91^*^	0.87^*^	−0.84^*^	1					
M	−0.89^*^	−0.98^*^	0.91^*^	−0.92^*^	0.91^*^	1				
PA of PMN	−0.88^*^	−0.95^*^	0.90^*^	−0.90^*^	0.84^*^	0.95^*^	1			
PA of M	−0.01	−0.11	0.06	−0.02	0.13	0.11	0.06	1		
NETs	−0.87^*^	−0.96^*^	0.90^*^	−0.92^*^	0.90^*^	0.96^*^	0.93^*^	0.10	1	
Cortisol	0.87^*^	0.89^*^	−0.84^*^	0.84^*^	−0.83^*^	−0.89^*^	−0.88^*^	−0.04	−0.84^*^	1

* *p < 0.01*.

*PMN, neutrophil; L, lymphocyte; M, macrophage; Seg PMN, segmented neutrophil*.

### Viability and PA of Milk Phagocytes

**Figure 4:**
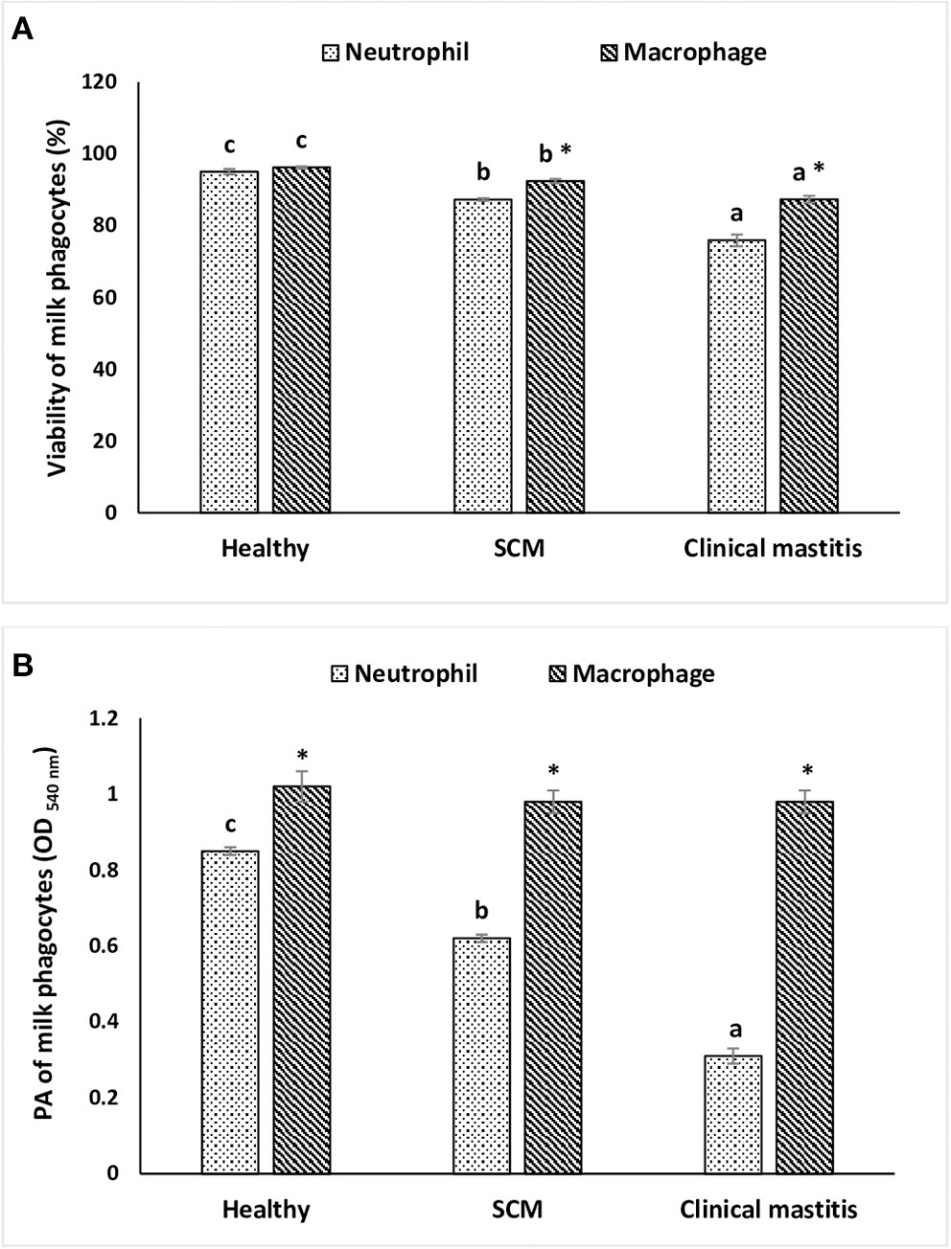
The viability **(A)** and *in vitro* phagocytic activity **(B)** of milk phagocytes in healthy, subclinical mastitis (SCM), and clinical mastitis Sahiwal cows. Data were expressed as mean ± SEM. Means statistically (*p* < 0.05) different between the groups are shown with superscript letters (a–c). *Statistical difference (*p* < 0.05) within the same group.

To investigate the impact of increased SCC and progress of mammary infections on the life span and activity of milk phagocytes, the viability and PA of neutrophils and macrophages were studied in healthy, SCM, and CM cows. The viability of milk phagocytes was highest in healthy milk (macrophage = 96.25% vs. neutrophils = 95.06%), decreased (*p* < 0.05) in the SCM group, and reached the lowest values in mastitis group (macrophage = 87.35% vs. neutrophils = 75.92%). Although the percentage of milk macrophages and neutrophils decreased dramatically in the mastitis group as compared to the healthy group, the decrease in the percentage of live cells was greater in neutrophils as compared to macrophages (Figure 4A). The PA of milk neutrophils was highest in the healthy group (0.85 ± 0.01), decreased in the SCM group, and continued to decline (*p* < 0.05), with the progress of mammary infection reaching the lowest values in the CM group (0.31 ± 0.03). Unlike neutrophils, the PA of milk macrophages remained unchanged in the SCM and CM groups as compared to the healthy group. Moreover, the PA of milk macrophage was higher (*p* < 0.05) than that of milk neutrophils in all the groups of cows (Figure 4B). The increase in the SCC and neutrophil percentage was negatively associated (*p* < 0.05) with the viability, PA, and NET formation of milk neutrophils (Table 2).

### NET Release by Milk Neutrophils

**Figure 5:**
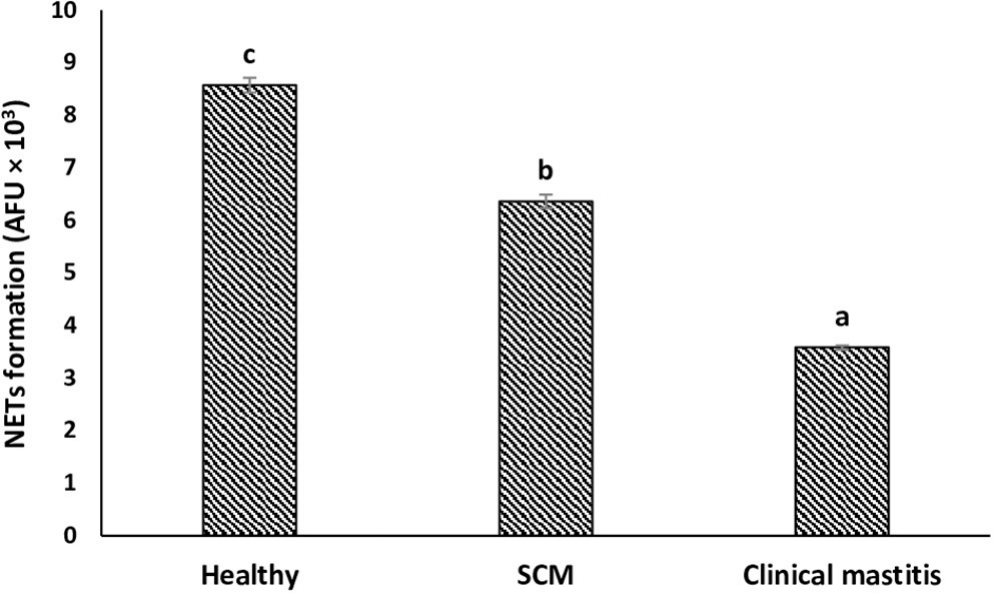
NET release by milk neutrophils of healthy, subclinical mastitis (SCM), and clinical mastitis Sahiwal cows. Extracellular DNA quantifications of supernatants from milk neutrophils following zymosan treatment (1 mg/mL, 3 h) using fluorometric DNA quantification assay (PicoGreen). Data were expressed as mean ± SEM. Means statistically (*p* < 0.05) different between the groups are shown with small superscript letters (a–c).

To explore the impact of mammary infection on the ability of milk neutrophils to release extracellular traps, NETosis was quantified using PicoGreen-derived fluorescence intensities. Measurement of the concentration of extracellular DNA (×10^3^ AFU) in the supernatant of milk neutrophils revealed NET formation in response to stimulation with zymosan. The concentration of extracellular DNA in milk neutrophils isolated from the healthy group of cows was the highest (8.57 ± 0.14). However, it decreased (*p* < 0.05) in the SCM group and continued to decrease with the progress of mammary infection reaching the lowest values (3.58 ± 0.04) in CM group (Figure 5).

### Expression of Different Receptors and Genes in Milk Neutrophils and Macrophages

**Figure 6:**
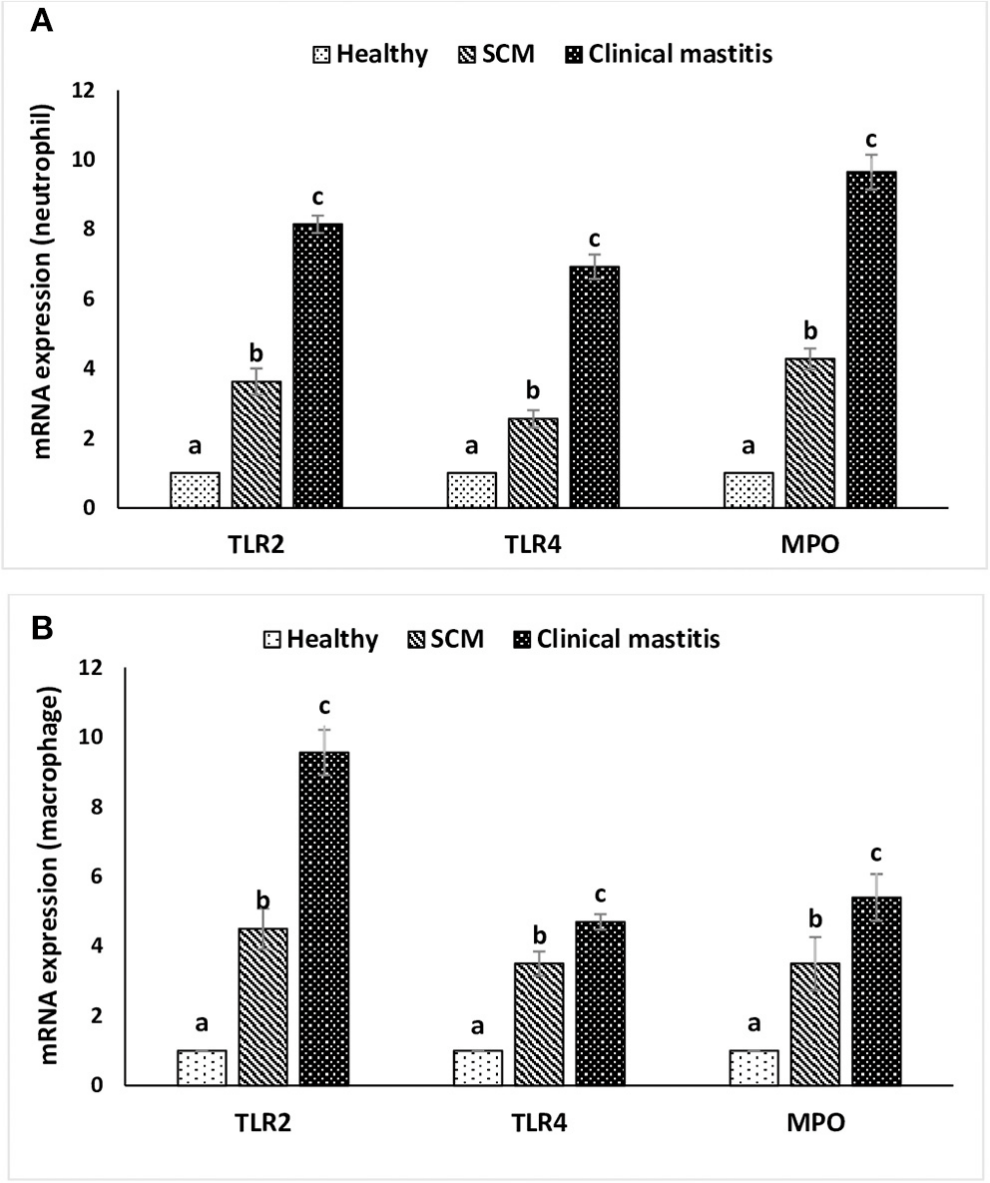
Relative mRNA expression of toll-like receptors (TLR2, TLR4) and myeloperoxidase (MPO) in milk neutrophils **(A)** and macrophages **(B)** of healthy, subclinical mastitis (SCM), and clinical mastitis Sahiwal cows. Data were expressed as mean ± SEM. Means statistically (*p* < 0.05) different between the groups are shown with small superscript letters (a–c).

**Figure 7:**
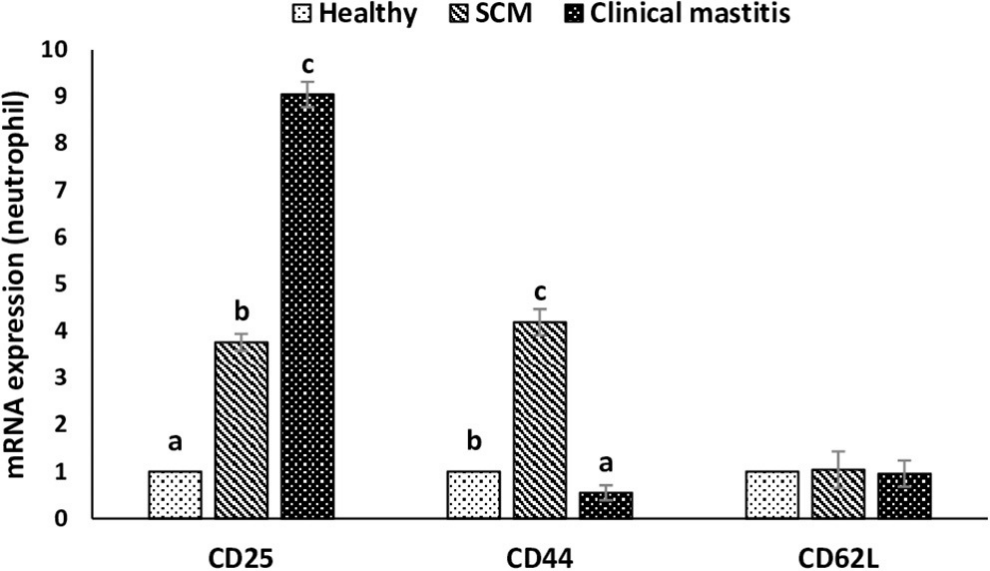
Relative mRNA expression of various cluster of designation receptors (CD25, CD44, CD62L) on milk neutrophils of healthy, subclinical mastitis (SCM), and clinical mastitis Sahiwal cows. Data were expressed as mean ± SEM. Means statistically (*p* < 0.05) different between the groups are shown with small superscript letters (a–c).

This experiment was to investigate the impact of different degrees of mammary infections on the expression of major genes and receptors essential for defense functions of milk phagocytes. The relative mRNA expression of TLR2, TLR4, and MPO has been presented in Figure 6A for milk neutrophils and in Figure 6B for milk macrophages. Relative mRNA expression of TLR2, TLR4, and MPO in milk neutrophils and macrophages was the lowest in the healthy group of cows. However, it increased (*p* < 0.05) significantly with the initiation of mammary infection in the SCM group and reached the highest values in the CM group. Although the relative mRNA expression of TLR2 was higher in milk macrophages compared to neutrophils, the mRNA expression of TLR4 and MPO was significantly higher (*p* < 0.05) in neutrophils compared to macrophages (Figure 6). The relative mRNA expression of CD molecules (CD25, CD44, CD62L) on milk neutrophils is presented in Figure 7. The relative mRNA expression of CD25 was lowest in healthy milk and increased (*p* < 0.05) with the development of mammary infection reaching the highest values in the CM group. The expression of CD44 increased (*p* < 0.05) during the initial stage of mammary infection in SCM. However, it decreased (*p* < 0.05) with the progress of mammary infection in the CM group as compared to the healthy group. Unlike all the previous genes and receptors, the relative mRNA expression of CD62L on milk neutrophils remained unaltered in all the studied groups of cows (Figure 7).

### Milk Plasma Cortisol

**Figure 8:**
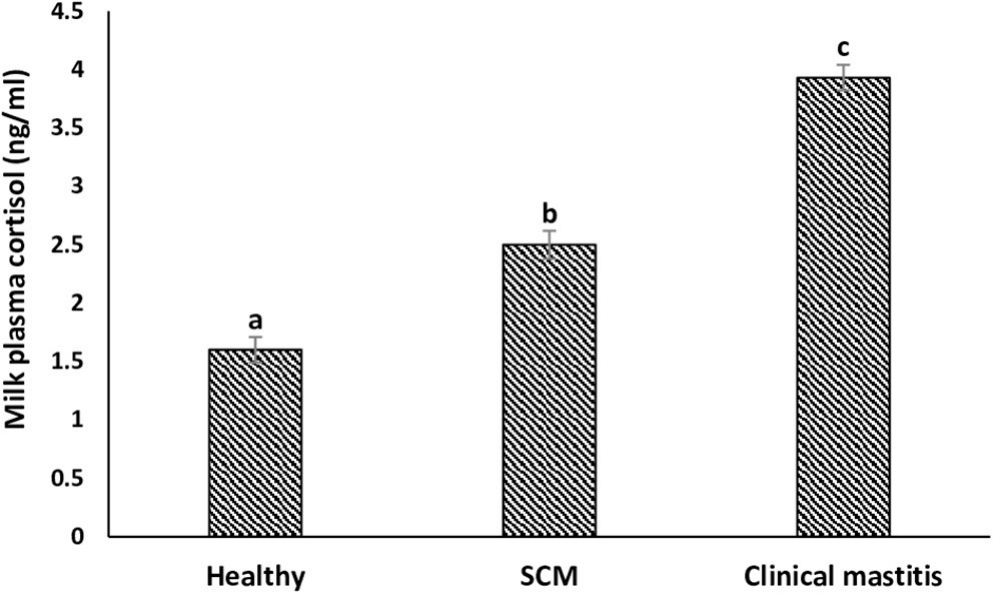
Milk plasma cortisol concentration (ng/mL) in healthy, subclinical mastitis (SCM), and clinical mastitis Sahiwal cows. Data were expressed as mean ± SEM. Means statistically (*p* < 0.05) different between the groups are shown with small superscript letters (a–c).

The concentration (in ng/mL) of milk plasma cortisol was minimum (1.60 ± 0.11) in the milk samples collected from the healthy group of cows. However, it started increasing (*p* < 0.05) during the subclinical form of mammary infection (2.50 ± 0.12) and continued to increase with the progress of mammary inflammation reaching the maximum values in the CM group (3.93 ± 0.11) (Figure 8).

## Discussion

Bovine mastitis, an inflammation of the mammary gland, is known to be the most significant disease in dairy cattle globally. It does not only affect animal health but also put a huge economic burden on the dairy industry due to milk production losses, low milk quality, and increased cost of treatments (3, 4). However, the best strategy to combat mastitis is its prevention from occurrence. Following some proper management practices in dairy farms, diagnosing mastitis at the earliest and understanding the underlying mechanism of mammary immune response to invading pathogens are some key aspects for prevention (21, 25, 31, 32). However, mastitis is usually caused by multiple infectious agents, and to fight against the infections, the mammary gland has a line of defense system called SCs, which comprise white blood cells and milk epithelial cells. Owing to a pathogenic insult, the number of SCs in milk increases, and monitoring of SCC (number of cells per mL of milk) helps in determining the mammary inflammation (4, 11).

In our study, we observed that a significant increment in SCC during udder inflammation with the highest number was recorded in CM cows. The reason for rising SCC might be a protective mean to kill the pathogens, and this is associated with the severity and duration of mammary inflammation (33). Nonetheless, to differentiate between healthy and mastitis milk, every country has set a limit for SCC, and in Indian condition, healthy mammary gland should have 150 × 10^3^ SCs/mL of milk, whereas a value higher than this denotes mammary health disturbance (4, 26). However, various factors could influence the count of SCs including the stage of lactation, parity, season, frequency of milking, and physiological and environmental stress (4, 27, 34). Moreover, diurnal variation in the milk SCC has been reported in cows and buffaloes (35, 36). Therefore, differential cell counts can be used as an alternative approach for a more detailed analysis of the health status of mammary gland (37, 38). DLC indicates the percentage of individual cell populations such as neutrophils, macrophages, and lymphocytes, which play critical roles during intramammary infection (39). Usually, healthy udder comprises predominantly macrophages, whereas elevated neutrophil percentage is characteristic of the infected udder (15, 38).

Macrophage and neutrophils have a common precursor of origin and known to be the professional phagocytes that work in a coordinated manner during an inflammatory immune response (40). Macrophages are sentinel cells that recognize the intruding pathogens and release various chemoattractants upon activation. The chemical messengers subsequently activate neutrophils and facilitate their transmigration from blood to the inflamed tissue (1, 4). This signaling cascade justifies the drastic rise in the number of milk neutrophils with the progress of mammary infection observed in our study. Paape et al. (1) also reported that neutrophils rapidly transmigrate to the inflamed mammary gland, and their number may go up to 90% during mastitis. In accordance with our findings, Gulbe et al. (41) demonstrated decreased percentage of milk macrophage in sub-clinically infected mammary glands. We speculate that the absolute number of milk macrophages did not change significantly as the decrease in their percentage may be attributed to the parallel increase in the number of neutrophils.

The severity of infection not only increases the rate of neutrophil production from bone marrow but also reduces its maturation time, and it leads to the left shift, that is, release of immature neutrophils/band cells (1). We found a higher percentage of band cells in mastitis cows in comparison to healthy and SCM cows. Nevertheless, the immature neutrophils cannot function normally as compared to mature neutrophils, which comprise the disease resistivity of infected animals. Also, the pathological condition of mammary gland influences the viability of neutrophil and delineates the tissue retention of neutrophil after recruitment (42). Lower viability and activity of milk phagocytes isolated from mastitis milk are correlated with the pathogens and their components, which are known to influence the life span and functions of milk phagocytes (43). The present study was limited to mastitis naturally induced by *S. aureus* as this pathogen is responsible for the majority of mastitis cases in the study area (44). In our previous study, we reported lower inflammatory responses and impaired neutrophil activities during Gram-positive bacterial infections (*S. agalactiae* and *S. aureus*) as compared to Gram-negative bacterial infections (*E. coli*), which may explain the association between *S. aureus* infection and chronic mastitis (17). Also, we reported pathogen-dependent modulation of neutrophil viability in which the viability of milk neutrophils decreased with the progress of mammary infection and reached the lowest values in *S. aureus*–infected cows compared to mastitis induced by *S. agalactiae* or *E. coli* (17).

The main function of neutrophils is to kill the invading pathogens, and neutrophils can do it through several effector mechanisms including phagocytosis, excretion of granules contents of antimicrobial peptides/proteins, and NETs (1, 23, 45). Phagocytosis involves the respiratory burst, and *in vitro* PA is an effective parameter to investigate when evaluating mastitis resistance (21). There was a significant decrease in the PA of milk neutrophils in mastitis group as compared to SCM and healthy groups. The reason might be due to the negative effect of cortisol on PA as the concentration of milk cortisol was the highest in mastitis cows. Cortisol exerts its pleiotropic effects via the cytoplasmic receptors, which are present in various immune cells, and one of the effects is impairment of reactive oxygen species production (18). In the present study, we observed no decrease in the PA of milk macrophages in association with the increased cortisol concentration and the decreased macrophage viability in SCM and CM milk samples. Previous studies showed that dexamethasone promotes phagocytosis and bacterial killing ability of monocytes/macrophages challenged with various pathogens, and the blocking of glucocorticoid receptor could impair their PA (46). This can partially explain the maintained level of PA of milk macrophage during mammary infection, but more studies are warranted to explore this mechanism in bovine mastitis.

MPO is one of the effector lysosomal enzymes present in the azurophilic granules of neutrophils, and it gets released by activated neutrophils during mammary infections (47). During neutrophils' respiratory burst, MPO mediates the transformation of hydrogen peroxide into hypochlorous acid. This process is essential during phagocytosis for efficient killing and elimination of microorganisms invading mammary tissue (45, 47). Moreover, MPO is one of the main proteins released during the extrusion of NETs by neutrophils. Although the expression of MPO increased in milk neutrophils isolated from SCM and CM cows, the PA and NET formation decreased in these cows. This is because phagocytosis and NET formation are multistep processes that involve many essential molecules and pathways, and the impairment of any step could impair the whole process. Although MPO was always studied in neutrophils, a recent study revealed that bovine monocytes are also expressing MPO comparable to neutrophils (48). In the present study, the expression of MPO in milk macrophages was comparable to milk neutrophils in healthy mammary gland and increased significantly in SCM and CM samples. However, the expression of MPO in neutrophils isolated from CM cows was double the expression of MPO in macrophages isolated from the same group. This indicates that this enzyme is more important for the functions of neutrophil especially NET release during mammary infections.

NETs are extracellular structures of DNA framework decorated with histones and many granular proteins including MPO and neutrophil elastase. This defense mechanism exhibited by neutrophils is critical for the control and elimination of pathogens (22–24). Recent studies have proved that milk neutrophils can perform NETs in response to mammary infections in sheep and cows (49, 50), but the variation in NET formation by bovine neutrophil in relation to changes in milk SCC and DLC has not been reported. The ability of milk neutrophils to perform NETs under *in vitro* condition was maximum in healthy cows in association with lower SCC and minimum cortisol concentration. However, the increases in SCC, neutrophil percentage, and cortisol concentration with the progress of mammary infection were associated with diminished NET formation. The decreased NET release by milk neutrophils of infected cows could be because these cells have already been subjected to *in vivo* activation, utilized part of their energy resources, and the increased percentage of immature band neutrophils. This also can explain the mitigated innate immune response and the longer time required to clear infection in SCM and CM cows.

TLRs are the pathogen recognition receptors that help in the recognition of a wide range of pathogen-associated molecular patterns and elicit the innate immune response. TLR2 and TLR4 are known to recognize different bacterial structures such as TLR2 ligands, that is, peptidoglycan of Gram-positive bacteria, and TLR4 ligands, that is, lipopolysaccharide of Gram-negative bacteria. Higher expression of TLR2 and TLR4 in neutrophil during mastitis might indicate higher recognition and elevated immune response against the microbes (49). We observed a significant increase in the expression of TLR2 and TLR4 in both neutrophils and macrophages with the progress of mammary infection. Griesbeck-Zilch et al. (51) reported that the expression of TLR2 significantly increases during mastitis induced by *S. aureus*, which facilitates effective recognition and elimination of the Gram-positive bacteria by the host immune cells. Underhill et al. (52) reported that TLR2 is recruited to macrophage phagosomes and discriminates between pathogens. Moreover, a point mutation in TLR2 abrogates the inflammatory responses to yeast and Gram-positive bacteria, but not to Gram-negative bacteria. However, a recent study reported that soluble TLR2 and full-length TLR2 are released by human macrophages in response to lipopolysaccharide challenge, which may contribute to glucocorticoids-induced immunosuppression and chronic infections (53). We observed that the expression of TLR2 was double the expression of TLR4 in both neutrophils and macrophages during mastitis, which reflects the role of TLR2 in mediating an effective immune response against *S. aureus*. However, the increase in the expression of TLR4 in milk phagocytes isolated from SCM and CM mastitis was also significant, which indicates that this receptor is also critical for the elimination of the invading pathogens. Similarly, Gonen et al. (54) reported that adoptive transfer of TLR4-expressing macrophage restricted the invasion of epithelial cells by *E. coli* in a murine model of acute mastitis.

CD25 is the main receptor for IL-2, and the higher expression of CD25 in bovine neutrophils was reported as a potential biomarker of inflammation during various diseases such as mastitis (55). IL-2 is a pro-inflammatory cytokine secreted by type 1 helper cells and amplifies the immune response against both Gram-positive and Gram-negative bacteria. Recently, we have reported increased concentrations of plasma IL-2 during mastitis induced by both *S. aureus* and *E. coli* (17). In the present study, the increased expression of CD25 is essential to mediate the signaling of IL-2 in milk neutrophil during mammary infection. Adhesion molecules such as selectin and integrins are necessary for effective neutrophil recruitment to the sites of inflammation. The very first step of recruitment is the capturing/tethering, which is mediated by the CD62L (l-selectin), which helps in slowing down of neutrophil and allowing the neutrophil to roll along with the vascular endothelial cell (56). However, for tight binding with the endothelium, integrin is required, and for that, neutrophil should shed CD62L after proteolytic cleavage (57). This might be the probable reason for the lower expression of CD62L observed in the milk neutrophils of all the groups.

CD44 is a non-specific accessory adhesion molecule that has been reported to play a critical role in mediating the trafficking of leukocyte to extra lymphoid sites of inflammation (58, 59). Higher expression of CD44 in milk neutrophils isolated from SCM cows may be important for the recruitment of neutrophils to the mammary tissue during the initial stage of infection. After neutrophils perform their functions at the site of infection, they get removed by macrophages, and CD44 serves as an apoptotic signal for the macrophages (60). Pathogen-dependent modulation of CD44 expression has been reported by Harp et al. (58), in which they found increased expression of CD44 on milk lymphocytes during *Streptococcus uberis* mastitis but not with *Serratia marcescens*. Lower expression of CD44 in neutrophils isolated from mastitis cows in our study has two explanations. First, neutrophils were isolated at the beginning of mastitis occurrence, and it could be too early for macrophages to start clearing neutrophils as the removal of apoptotic neutrophils occurs after the inflammation subsides. Second, it could be induced by *S. aureus* to impair CD44 signaling and the recruitment of immune cells, which leads to mammary tissue injury and chronic inflammation usually observed in mastitis caused by this pathogen. Similarly, Cairns et al. (61) demonstrated that decreased expression of CD44 on neutrophils and monocytes during systemic lupus erythematosus impairs neutrophil clearance by macrophage and increases disease severity.

## Conclusions

The present study highlights the importance of SCC and DLC as novel methods for monitoring the health status of mammary gland in dairy cows. DLC reveals the alteration in milk leukocyte population without an increase in total cell number and thus could identify infected quarters more efficiently than SCC. The increase in the SCC and percentage of milk neutrophils during subclinical and clinical forms of mammary infection was associated with reduced phagocytosis and extracellular trap formation. Milk cortisol was positively correlated with the number of milk neutrophils and expression of TLRs, MPO, and CD25, whereas it was negatively correlated with the PA of neutrophils coming in milk. This may increase the chance of CM in cows; however, this interplay needs to be explored further in a large number of animals particularly with respect to the functions and gene expression of milk phagocytes. This is essential to improve animal welfare and increase farmer income as well as milk quality and production.

## Data Availability Statement

The original contributions presented in the study are included in the article/supplementary material, further inquiries can be directed to the corresponding authors.

## Ethics Statement

The animal study was reviewed and approved by the approval of all the experiments carried out in this research work was obtained from the Animal Ethics Committee of the NDRI according to the CPCSEA rules, laid down by the Government of India.

## Author Contributions

MA and AD designed the study. MA performed all the experiments, analyzed the data, prepared the figures, wrote, and revised the manuscript. AD supervised the project and provided the funds. BSKP helped in sample collection and wrote some of the paper. All authors read and approved the final version of the manuscript.

## Conflict of Interest

The authors declare that the research was conducted in the absence of any commercial or financial relationships that could be construed as a potential conflict of interest.
